# Self-assembled 2D WSe_2_ thin films for photoelectrochemical hydrogen production

**DOI:** 10.1038/ncomms8596

**Published:** 2015-07-01

**Authors:** Xiaoyun Yu, Mathieu S. Prévot, Néstor Guijarro, Kevin Sivula

**Affiliations:** 1 Laboratory for Molecular Engineering of Optoelectronic Nanomaterials, Institute of Chemical Sciences and Engineering, École Polytechnique Fédérale de Lausanne (EPFL), Station 6, CH H4 565, Lausanne 1015, Switzerland

## Abstract

WSe_2_—a layered semiconductor that can be exfoliated into atomically thin two-dimensional sheets—offers promising characteristics for application in solar energy conversion. However, the lack of controllable, cost-effective methods to scalably fabricate homogeneous thin films currently limits practical application. Here we present a technique to prepare controlled thin films of 2D WSe_2_ from dispersions of solvent-exfoliated few-layer flakes. Flake self-assembly at a liquid/liquid interface (formed exceptionally from two non-solvents for WSe_2_) followed by substrate transfer affords large-area thin films with superior 2D flake alignment compared with traditional (liquid/air) self-assembly techniques. We further demonstrate, for the first time, solar-to-hydrogen conversion from solution-processed WSe_2_ thin films. Bare photoelectrodes with a thickness of *ca.* 25 nm exhibit sustained p-type photocurrent under simulated solar illumination, and up to 1.0 mA cm^–2^ at 0 V versus reversible hydrogen electrode with an added water reduction catalyst (Pt). The importance of the self-assembled morphology is established by photoelectrochemical and conductivity measurements.

## Abstract (web summary)

Practical application of 2D transition metal dichalcogenides is limited by the lack of scalable fabrication methods. Here, the authors present a technique to prepare controlled thin-films of 2D WSe_2_ from dispersions of solvent-exfoliated flakes and demonstrate their use for solar water reduction.

## Graphical abstract

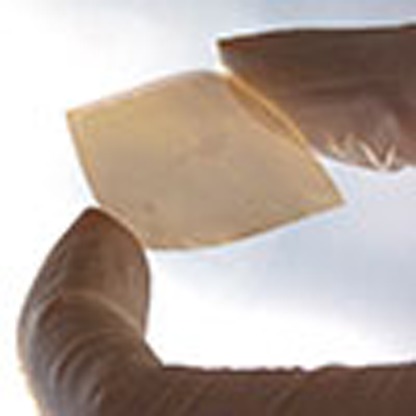

Transition metal dichalcogenides (TMDs) with a layered crystal structure have recently gained extensive attention due to their ability to be exfoliated into atomically thin two-dimensional (2D) sheets. The unique mechanical, physical and chemical properties of these 2D TMDs have established them as promising candidates for high-performance flexible electronic and optoelectronic devices^1^. In particular, the semiconducting MoX_2_ or WX_2_ (*X*=S or Se) show tunable band-gap characteristics corresponding to the number of atomic layers^2^, and strong light-matter interactions that afford up to 10% absorption of incident solar illumination in a thickness of <1 nm—capturing sunlight stronger than common semiconductors (that is, GaAs and Si) by an order of magnitude^3^,^4^. Moreover, early studies on bulk (non-exfoliated) TMDs for solar energy conversion achieved impressive results. Notably, WSe_2_-based Schottky junctions achieved 8.6 (ref. 5) and 14% (ref. 6) energy conversion efficiency, respectively, in photovoltaic (PV) and photoelectrochemical (PEC) devices. Accordingly, outstanding performance in solar energy conversion with 2D TMD materials has been greatly anticipated.

To take advantage of the 2D nature of TMDs in solar applications, the well-known mechanical exfoliation technique (that is, the scotch tape method) has been recently employed. Micrometre-sized van der Waals p–n heterojunction devices have been constructed by few-layer MoS_2_ and WSe_2_, and have been observed to be photoactive^7^,^8^. However, the further development of solar energy conversion devices using these materials, and their ultimate practical application, is hindered by the lack of a controllable method to fabricate large-area and continuous thin films with controllable layer orientation and suitable contact to a conducting substrate. While physical or chemical vapour deposition techniques can offer good control over thin-film preparation^9^,^10^, these methods require high temperatures and vacuum, which are less suitable for the economical production of large-area solar energy conversion devices. Solution-based processing methods, in contrast, offer a viable route. Indeed, the chemical exfoliation of TMDs (using lithium intercalation) can afford stable dispersions of 2D flakes suitable for controllable film formation^11^. However the concurrent change from the semiconducting 2H-phase to the metallic 1T-phase during chemical exfoliation causes a loss in photo-activity^12^,^13^. Alternatively solvent-assisted exfoliation^14^,^15^,^16^ can yield semiconducting 2D flakes, though due to the defect-free flake surfaces produced, obtaining stable dispersions at high concentration and processing them into uniform thin films without layer restacking remain significant challenges. We recently reported a sonopolymer-assisted exfoliation method that, together with alkyl-trichlorosilane surfactants, gave extremely stable dispersions of high-concentration 2D MoS_2_ in 1,2-dichlorobenzene (DCB), and showed promise for thin-film formation^17^. Nevertheless, the development of deposition methods that can offer precise control over film thickness and morphology from TMD dispersions remains essential for further implementation into solar energy conversion devices.

Here we present a novel method for the controlled processing of 2D WSe_2_ dispersions into uniform large-area thin films with flakes oriented either edge-to-edge (parallel to the substrate) or in overlapping-aggregated morphologies. We leverage this method to demonstrate, for the first time, solar-to-hydrogen energy conversion from solution-processed WSe_2_ thin films, and further gain insight into the importance of the film morphology on the photoelectrode performance.

## Results

### Flake exfoliation self-assembly and film deposition

Generally in solution-based nanoparticle thin-film formation, precipitation and aggregation lead to poor uniformity and inadequate contact with the substrate. While partial control over these aspects can be achieved using appropriate surfactants or nanoparticle surface ligands, conventional solution-based coating techniques (drop casting, spin coating or dip coating) still typically give rise to agglomerated structures after solvent evaporation, especially for 2D materials^18^. As such, much work has focused on applying the 2D template of a fluid/fluid interface for the assembly of nanomaterials, followed by transfer to a solid substrate to achieve thin-film formation in a controlled manner. The self-assembly of nanoparticles at a liquid/air interface—the so-called Langmuir–Blodgett technique—is the prototypical example of this approach^19^,^20^, and employing a liquid/liquid interface is a promising extension suitable for large-aspect ratio 2D materials^18^. Typically in liquid/liquid self-assembly techniques, nanoparticles dispersed in a hydrophilic phase are driven to a hydrophilic/hydrophobic interface using mechanical agitation or with an inducing agent^18^,^21^,^22^. For example in seminal work by Vanmaekelbergh and co-workers^23^, ethanol was used as an inducer for the self-assembly of gold nanoparticles at a water–heptane interface. Recently, Jia *et al.*^24^ presented a similar approach with 2D sulphonated graphene by first dispersing the particles in the inducing solvent (ethanol) and injecting them into the water layer. However, solvent-exfoliated semiconducting TMD flake dispersions show poor stability in most solvents. Indeed, our attempts to apply traditional liquid/liquid self-assembly techniques were unsuccessful even with alkyl-trichlorosilane functionalized TMDs, as we observed aggregation during the flake migration and film organization processes rather than homogeneous film formation. Therefore, to enable the self-assembly of 2D TMDs we developed an approach to spatially confine the particles at the interface of two immiscible non-solvents for the TMD flakes, that is, ethylene glycol (EG), and hexane.

To demonstrate this deposition approach, we chose WSe_2_ as a model TMD given its previously demonstrated performance as a non-exfoliated semiconductor in solar energy conversion devices. Accordingly, we began by extending our previously reported method for the sonopolymer-assisted exfoliation of MoS_2_ to WSe_2_, demonstrating the generality of this approach^17^. Indeed a similar 2D sheet size (200–1,000 nm) and thickness (monolayer and few layer flakes) were obtained by exfoliating the bulk WSe_2_ in DCB as described in the methods section.

**Figure 1:**
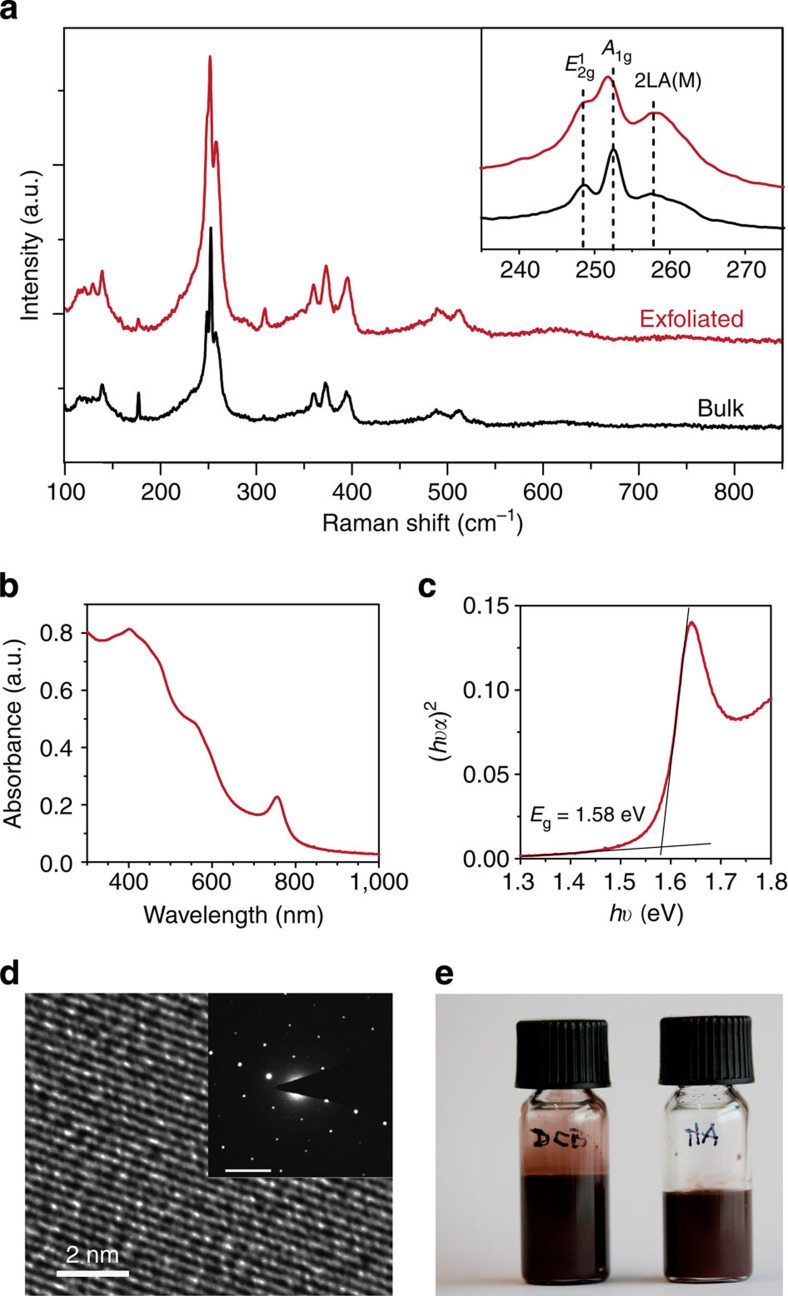
Basic characterization of the sonopolymer-assisted solvent-exfoliated WSe_2_. (**a**) Survey and detailed (inset) Raman spectra of bulk WSe_2_ and exfoliated WSe_2_ after the washing process. (**b**) UV–vis absorption spectrum of WSe_2_ dispersion in DCB and (**c**) the Tauc plot. (**d**) High-resolution-TEM image and SAED pattern (inset) of a single WSe_2_ flake after solvent assisted exfoliation, scale bar in SAED, 5 nm^–1^. (**e**) A photograph of the WSe_2_ dispersion in DCB solvent after 3 months (left) and WSe_2_ dispersion after solvent exchange with hexlyamine (right).

To verify the exfoliation of the bulk WSe_2_, basic characterization was preformed (Fig. 1). First, exfoliated material was compared with the bulk starting material by Raman spectroscopy (Fig. 1a). For the starting material, the peaks corresponding to the first order *E*_2g_ and *A*_1g_ Raman modes, respectively^25^, are clearly distinguished ∼250 cm^–1^ with a frequency difference of 4.0 cm^–1^ (inset Fig. 1a). In contrast for the exfoliated sample, these modes are merged into one peak with a shoulder (frequency difference of 2.8 cm^–1^). This decrease in frequency difference, caused by the reduced influence of neighbouring layers, confirms the effective exfoliation of the WSe_2_ into a 2D configuration^26^. Moreover, a weak Raman signal at 308 cm^–1^ in the exfoliated material suggests that the product contains mostly few-layered WSe_2_ (ref. 27), even though we observe the presence of some monolayer flakes by scanning electron microscopy (SEM). Importantly, no peak at 700 or 800 cm^–1^ was observed, which would indicate a substantial formation of oxide^28^. To gain further insight in to the quality of the WSe_2_, W core level XPS spectra were analysed and the W peaks (Supplementary Fig. 1) match well with the bulk material^29^. The doublet peaks at 36 and 38 eV can be attributed to a small amount of W-O in exfoliated sample similar to other reports^30^. However, we note that the broad 5P_3/2_ peak also is observed in the range of the oxide peaks and this was not considered in our fitting.

High-resolution TEM imaging of a single exfoliated WSe_2_ flake (Fig. 1d) shows continuous lattice fringes demonstrating the single crystal nature of the exfoliated WSe_2_. This is supported by selective area electron diffraction (SAED, Fig. 1d inset), which shows the hexagonal crystal structure corresponding to the 2H phase of WSe_2_. The UV–vis spectrum of the exfoliated WSe_2_ (Fig. 1b) shows several transition modes and an overall onset of *ca.* 800 nm. A direct band gap was estimated to be 1.58 eV by Tauc plot analysis (Fig. 1c), which is blue-shifted compared with the bulk material due to confinement effects^31^. The final dispersion of the exfoliated, hexyl-trichlorosilane (HTS) capped WSe_2_ in DCB at high concentration (*ca.* 25 mg ml^–1^, Fig. 1e) was found to be stable for several months. We note that it was also possible to create stable dispersions of the WSe_2_ in other solvents by adding the desired solvent after the washing procedure. Indeed, to transport the 2D WSe_2_ flakes to the aforementioned EG/hexane liquid/liquid interface for self-assembly, the exfoliation solvent DCB is not suitable given its high density. Thus, hexylamine was selected as an alternative solvent due to its intermediate density, miscibility with hexane and suitability to disperse the HTS functionalized 2D WSe_2_ (at 10 mg ml^–1^ a dispersion was found to be stable for hours).

**Figure 2:**
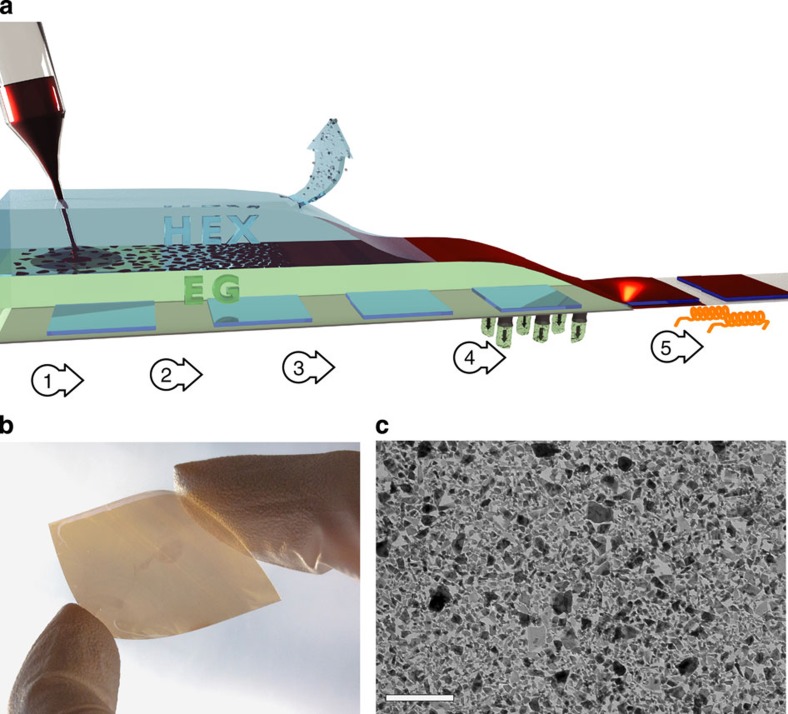
The space-confined self-assembly approach for 2D TMD thin-film deposition and results with WSe_2_. (**a**) shows a schematic of the deposition method as described in the main text. Step 1: injection of WSe_2_ dispersion; Step 2: flake confinement and self-assembly; Step 3: hexane removal; step 4: ethylene glycol removal and film deposition; step 5: drying at 150 °C. (**b**) displays a photograph of a single-flake-layer WSe_2_ thin film deposited on flexible Sn:In_2_O_3_ (ITO)-coated PET plastic, and (**c**) shows a representative TEM image of a single-flake-layer WSe_2_ layer SCSA thin film deposited on a carbon-coated TEM grid. The scale bar is 2μm.

Our space-confined self-assembled (SCSA) thin film deposition method proceeds as displayed in Fig. 2a. First, the WSe_2_ dispersion is injected into the hexane layer near the EG/hexane interface. As the hexylamine rapidly dilutes into the hexane layer, the flakes become confined by the two non-solvents and quickly align with the 2D interface. Increasing the loading of flakes leads to in-plane self-compression and generates a compact 2D self-assembly of the TMD. Next, the hexane layer is removed from the top leaving the self-assembled thin-film floating on the surface of the EG. The EG can then be removed from the bottom (through fritted glass) affording the deposition of the SCSA WSe_2_ film on a chosen substrate (for example, conductive F:SnO_2_-coated glass, SiO_2_/silicon or flexible Sn:In_2_O_3_-coated polyethylene terephthalate, PET, plastic). Finally, the film is dried at 150 °C to remove any residual EG and improve film adhesion.

Unlike conventional liquid/liquid interfacial self-assembly techniques, in which the solubility of the nanoparticles in one of the phases can allow folding and overlapping of the 2D structures, the two non-solvents employed in our approach provide much stronger confinement to the WSe_2_ flakes at the interface. The effective use of the two non-solvents has been furthermore enabled by the high-concentration and stable TMD dispersion afforded by the exfoliation method and subsequent HTS functionalization. This allows only a small volume of the dispersion to be used maintaining the hexane phase a non-solvent. In addition for our approach, we note that overloading the flakes at the EG/hexane interface leads only to local small-area aggregates without affecting the entire film. This allows the facile and rapid preparation of large-area close-packed films of a single layer of flakes. Accordingly, the thickness of the SCSA film is insensitive to the processing conditions and determined by the flake thickness. Thicker films can be obtained by repeating the deposition process in a layer-by-layer strategy. A typical single flake layer SCSA film of WSe_2_ on flexible Sn:In_2_O_3_-coated PET substrate is shown in Fig. 2b, and is homogeneous over a large area (7.5 cm^2^). Notably, this method is straightforward to scale up and homogeneous films up to 22.5 cm^2^ have been prepared (see Supplementary Fig. 2). Transmission electron microscopy (TEM; Fig. 2c) provides further detail of the morphology of the horizontally oriented thin film. No significant aggregation or restacking of the flakes is observed due to the 2D alignment afforded by the SCSA film formation technique. We note that, due to the irregular shape of the solvent-exfoliated flakes, some spaces between flakes are present. Moreover, we can roughly estimate that the HTS remaining in the SCSA-deposited films is in the order of 10 mol% (that is, 1 HTS molecule for every 10 W atoms, see methods section).

To further illustrate the importance of the spatial confinement in the thin film formation process, we prepared WSe_2_ thin films by injecting the hexylamine flake dispersion on the EG surface without a hexane top layer. As the hexylamine evaporates, we observe the partial restacking of the WSe_2_ flakes indicated by a colour change and a relatively reduced lateral coverage of material on EG surface. After complete hexylamine removal, the aggregated WSe_2_ film can be transferred to a substrate using the Langmuir–Blodgett technique, wherein the distance between WSe_2_ aggregates is minimized by film compression. We note that the material consumption in this aggregated–compacted (AC) film formation approach is *ca.* 15 times higher than in the SCSA approach to obtain similar lateral surface coverage on the substrate. A video recording demonstrating the clear difference between the SCSA and AC assembly process at the 2D interface is included as Supplementary Movie 1.

### Morphological comparison and film conductivity

**Figure 3:**
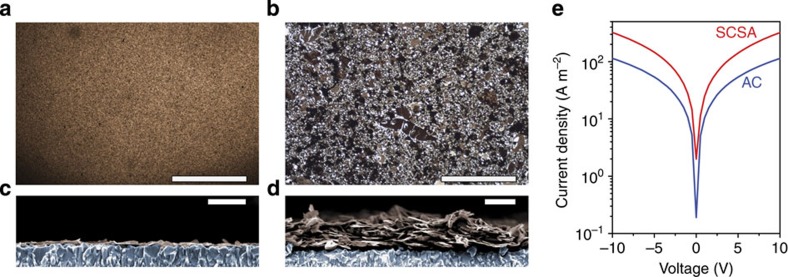
Morphological and electronic comparison of the SCSA and AC films. (**a**,**c**) show diascopic optical microscopy and cross-sectional SEM images, respectively, for the SCSA film while (**b**,**d**) are for the AC film. Scale bars in (**a**,**b**), 500 μm; and scale bars in **c**,**d**, 400 nm. The SEM images are coloured to highlight the F:SnO_2_ substrate (blue) and the WSe_2_ (brown). (**e**) shows the *J*–*V* data for the conductivity measurement on the 20 μm channel device as described in the main text.

The difference in morphology between the SCSA film and the AC film is shown with top-view (diascopic) optical microscopy and cross-sectional SEM images in Fig. 3. A homogeneous morphology is evident for the SCSA film on the millimetre length scale (Fig. 3a). Differences in the number of atomic layers in each flake and small spaces between the individual flakes do, however, give rise to some optical graininess on the 500 nm scale. In stark contrast, the AC film (Fig. 3b) exhibits domains in the 10–100 μm range and large void spaces despite the compression of the film before deposition. While some areas of the AC film appear to have similar optical transmission as the SCSA film, other areas are considerably more opaque, suggesting a widely varying thickness of WSe_2_ in this film. The cross-sectional images (Fig 3c,d) confirm that the film consists of a single flake layer and that flake orientation is parallel with the substrate in the case of the SCSA film, while much thicker films and more random flake orientation is observed in the AC film. We note that some roughness of the SCSA film is expected due to underlying substrate (F:SnO_2_-coated glass), and surprisingly despite regions of significant aggregation in the AC film and overlapping flakes (see additional SEM images in Supplementary Fig. 3), much of the film is reasonably aligned with the substrate.

While the SCSA film deposition technique clearly affords significantly more homogeneous films with improved flake alignment compared with the liquid/air technique, the resulting edge-to-edge configuration potentially leads to poorer charge transport in the direction parallel to the substrate (compared with the AC film case where flake overlap can facilitate charge transport). To investigate this, two-electrode conductivity measurements over 20 or 60 μm channels were performed on SCSA and AC films deposited on glass substrates. The resulting current density–voltage (*J*–*V*) curves (Fig. 3e and Supplementary Fig. 4) indicate that, despite the edge-to-edge configuration in the SCSA film, long range conductivity remains possible (corresponding to conductivity in the order of 10^–4 ^S m^–1^). We note that the similar values found with 20 and 60 μm channel lengths suggests the contact resistance between the gold electrodes and the WSe_2_ film can be considered negligible, see Supplementary Table 1. Remarkably, the AC film shows similar, but slightly lower, current density in the direction parallel to the substrate despite the film having an average thickness of 150 nm (as measured by profilometry), that is, more than 10 times the SACA film. Indeed, a current density of an order of magnitude greater than that observed in the SCSA film would be expected in the AC film given its thickness, if the connectivity of the film was similar over the channel length. Thus the observed current density in the AC film implies that the charge transport is limited by the large void spaces between aggregates on the tens of micron length scale, which reasonably reduces the number of continuous charge carrier percolation pathways in the AC film. However, we cannot discount differences in conductivity caused by the increased requirement for carriers to hop between WSe_2_ layers in the AC film morphology. Indeed it is well-known that that carrier mobility in layered 2D TMDs is much higher in the lamellar plane compared with the out-of-plane direction^32^. In any case, the self-assembly afforded by our SCSA film formation technique is clearly beneficial for lateral charge transport over large distances.

However, while the gaps between the aggregates may limit horizontal conductivity in the AC film, we cannot discount the possibility that its aggregated morphology could provide advantages in specific applications where high surface-area semiconductor junctions are beneficial—for example. electro and PEC applications. Indeed, as illustrated by the SEM images, the aggregates in the AC films offer significantly increased surface roughness compared with the SCSA film, therefore a larger semiconductor–electrolyte junction and more efficient charge transfer could reasonably be expected^33^. Moreover, the observed morphology of the AC film, consisting of a certain number of vertically orientated flakes, may benefit charge transport towards the substrate, aided by the fast in-plane charge mobility in 2D WSe_2_^32^. Thus while the SCSA and AC films give drastically different film morphologies resulting in disparate charge transport in the direction parallel to the substrate, it is not immediately clear which would be better suited for applications as large-area electrodes for energy conversion.

### PEC characterization

**Figure 4:**
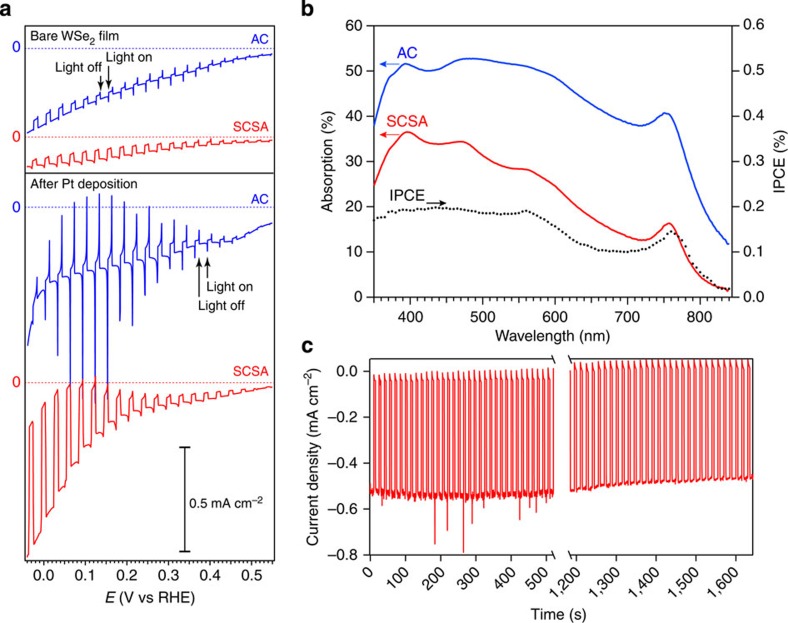
Photoelectrochemical characterization of the WSe_2_ thin films prepared by the SCSA and AC techniques. (**a**) shows the linear scanning voltammetry (LSV) curves under intermittent illumination (1 sun, 100 mW cm^–2^) for the bare films (top) and after Pt deposition (bottom). A horizontal axis corresponding to zero current is indicated for each curve. The incident-photon-to-current (IPCE) spectrum for the SCSA film is shown together with the optical absorptivity in **b**. (**c**) shows the chronoamperometric scan (at 0 V versus RHE) for the SCSA electrode under intermittent illumination.

To further examine the electronic behaviour of the WSe_2_ films, the PEC performance of the films deposited on conductive substrates were next assessed. In previous work, non-exfoliated (bulk) WSe_2_ was primarily investigated as an n-type photoanode for PEC oxidation reactions^5^,^6^. Indeed, its favourable light absorption and reported stability under oxidative conditions made it an attractive semiconductor for PEC energy conversion using aqueous polyhalide-based redox systems^34^,^35^. More recently, Lewis and co-workers^36^ reported PEC solar fuel production with p-type (Nb-doped) WSe_2_ bulk single crystals as photocathodes for hydrogen evolution via water reduction. While bare WSe_2_ electrodes exhibited negligible hydrogen production under illumination when polarized at potentials more positive than the reversible hydrogen electrode (RHE) reference potential, hydrogen evolution was observed from a catalyst (Pt/Ru)-coated WSe_2_ electrode in acidic electrolyte with a (1 sun) photocurrent density of 24.5 mA cm^–2^ at 0 V versus RHE. We examined our SCSA and AC electrodes (glass/F:SnO_2_/WSe_2_) under similar conditions and observed p-type behaviour without any intentional dopant added (consistent with undoped WSe_2_ measured by other techniques^37^,^38^). However, we cannot discount the HTS surfactant acting as a dopant^39^. The PEC performance is presented in Fig. 4. SCSA electrodes prepared from two layers were used to increase the light absorption for the PEC study. Despite an increased average thickness (25–30 nm for the two-layer case) the morphology was not different from the one-layer case (see Supplementary Fig. 5).

Linear scanning voltammetry (LSV) curves of the bare SCSA and AC electrodes were investigated under intermittent (chopped) illumination (1 sun, 100 mW cm^–2^) in 1 M H_2_SO_4_ electrolyte are shown in Fig. 4a (top section). Here we note the onset of sustained p-type (cathodic) photocurrent at *ca.* +0.5 V versus RHE for both the SCSA and AC electrodes. The magnitude of the photocurrent was also similar for both—reaching a maximum of *ca.* 40 μA cm^–2^ at 0 V versus RHE. We note that LSV measurements under chopped illumination show high dark currents due to transient effects. LSV measurements under constant dark or illumination conditions (Supplementary Fig. 6) confirm the dark current of the WSe_2_ films is similar to the bare F:SnO_2_ substrate and also corroborate the photocurrent magnitude. The observation of sustained photocurrent is remarkable with the bare films considering the absence of photocurrent in the single crystal case under the same conditions^36^. It is likely that the reasonably high density of edge states in our films directly act as catalytic sites for the hydrogen evolution reaction (HER) as these edge sites have been shown to be catalytically active for the HER in analogous TMD materials^40^,^41^. Comparing the photon absorption and incident-photon-to-current (IPCE) spectra as a function of the illumination wavelength, *λ*, confirms that the observed cathodic photocurrent results from light absorption by the WSe_2_ (Fig. 4b). Indeed, the IPCE matches well with the absorption spectrum from the onset at *λ*=800 nm to about 550 nm where it then remains roughly constant until *λ*=400 nm. This leveling off of the IPCE along with the modest photocurrent compared with the amount of light absorbed by both the SCSA and AC films suggests that factors other than light absorption limit the photocurrent of the bare films.

We next deposited a Pt catalyst to facilitate the HER and to gain more insight into the limitations of the WSe_2_ electrodes. Surprisingly, we found significantly different behaviour between the SCSA and the AC films (Fig. 4a bottom section). While the magnitude of the sustained photocurrent was similar to the bare film in the AC case, large transient photocurrent spikes were observed on illumination, which quickly decayed to steady-state photocurrent in the 50 μA cm^–2^ range. In contrast, the steady-state photocurrent of the SCSA electrode increased dramatically. The average photocurrent (out of eight replicate electrodes tested) at 0 V versus RHE for the Pt functionalized two-layer SCSA electrodes was 0.78±0.17 mA cm^–2^ and the highest measured was over 1.0 mA cm^–2^ (see Supplementary Fig. 7). To verify that the steady-state photocurrent was indeed due to hydrogen evolution in these electrodes, we measured H_2_ by gas chromatography (GC) under constant illumination and detected 103% of the hydrogen expected (compared with the photocurrent measured, See Supplementary Fig. 8), which is within the estimated 10% measurement error for a unity Faradaic efficiency of the HER on the SCSA electrodes. Moreover, similar to bulk single crystals, we observe good photoelectrode stability. Under constant polarization and intermittent illumination, insignificant change in the photocurrent was observed for tens of minutes (Fig. 4c). While longer stability measurements were not pursued in this work, the detachment of the Pt catalyst may decrease the photocurrent gradually. Despite this, the p-type WSe_2_ itself has been shown to have a similar long-term stability as the n-type photoanodes^36^.

## Discussion

The greatly improved PEC performance of the Pt-functionalized SCSA electrode compared with the AC electrode is surprising given the increased light absorption and surface area for charge transfer from the semiconductor to the electrolyte in the AC film case, as suggested by the observed morphology. Given the different SCSA and AC film thicknesses, (25 nm and 150 nm, respectively, as measured by SEM and profilometry), one possible explanation for the observed difference is poor charge transport through the WSe_2_ film in the AC film case. However, we eliminate this possibility by comparing the LSV curve for the AC electrode obtained from substrate-side illumination to the LSV from electrolyte-side illumination (See Supplementary Fig. 9). While the thickness of the AC film together with Beer–Lambert law indicates that 74% of the light absorption occurs in the first half of the film, equal performance was observed for both substrate-side and electrolyte-side illumination, suggesting that photogenerated carriers produced in high proximity to the charge collecting substrate do not have an increased collection efficiency compared with charges generated far from the substrate. This observation and the presence of the aforementioned large photocurrent transients in the LSV of the AC film suggest that charge carrier accumulation is occurring at the semiconductor/substrate interface or the semiconductor/electrolyte interface. The similar catalyst deposition conditions used for both films further suggests the semiconductor/substrate interface as the limiting factor of the AC films. Indeed, we propose that the excellent 2D self-assembly enabled by the SCSA film deposition technique supports an improved semiconductor/substrate contact compared with the AC case, and thus enables the significantly improved steady-state solar-to-hydrogen conversion. We note that further characterization is required in order to more fully understand the differences in the film formation, flake connectivity and electronic interfaces on the nanometre length scale for both the of the deposition methods. These detailed studies are underway in our laboratories.

In a broader perspective, the *ca.* 1.0 mA cm^–2^ hydrogen evolution photocurrent exhibited by the SCSA WSe_2_ films in this work is much lower than the 24.5 mA cm^–2^ obtained with optimized single crystal WSe_2_ together with Ru/Pt catalyst^36^. However, the ability to easily prepare large-area substrates, and to absorb a significant fraction of the solar spectrum using films only ∼10-nm thick are great advantages. Moreover our results, which clearly indicate the importance of HER catalysis and the semiconductor/substrate interface, offer points-of-interest to improve the performance of these solution-processed WSe_2_ photocathodes for the ultimate integration in PEC tandem cells for high efficiency solar hydrogen production^42^. Further development of underlayers and catalysts for this system are indeed underway in our laboratories. In addition, we suggest that the PEC study of solvent-exfoliated TMD materials, enabled by our material processing techniques, can be a valuable tool to aid the understanding and improvement of charge transport and transfer in these materials for application in PV and other optoelectronic devices. The extension of our methods to other TMDs and additional devices is also under development.

In summary, in this work we have presented for the first time, PEC water reduction from solution-processed WSe_2_ thin-film electrodes. Remarkably, bare WSe_2_ films showed measurable steady-state photocurrents without added catalyst. The further functionalization of these films with Pt gave hydrogen evolution photocurrents over 1.0 mA cm^–2^ under standard illumination conditions. This demonstration was enabled by the development of a novel SCSA approach for solvent-exfoliated 2D WSe_2_ flakes. The use of a liquid/liquid interface formed from two non-solvents for the WSe_2_ enabled strong confinement and superior self-assembly compared with a traditional (liquid/air) interfacial self-assembly technique. We showed that the improved contact with the conducing substrate afforded by our self-assembly technique (compared with films prepared by a liquid/air self-assembly technique) directly leads to an improved steady-state solar-to-hydrogen conversion, and highlights the importance of film morphology on photoelectrode performance. Given that our SCSA deposition technique is suitable for the roll-to-roll fabrication of large-area thin films^43^, we expect that it will be useful in the facile preparation of thin film electrodes from other TMD and layered materials. Finally, given the initial results presented here with 2D WSe_2_ and its promising aspects for solar fuel production, we anticipate future advances with this material deposited using solution-based self-assembly approaches.

## Methods

### Exfoliation of WSe_2_

In a typical exfoliation of WSe_2_, commercially available powder (AlfaAesar,<10 μm, 1.0 g) was dispersed in *ca.* 100 ml DCB. After sonication for 10 h in 0 °C bath (Qsonica Q700 probe sonicator, 50% amplitude, 5 s/1 s on/off cycles) large unexfoliated particles were removed by centrifugation (relative centrifugal force=2,000*g*, 30 min). HTS (0.5 v/v %) was then added into the supernatant to avoid flake restacking. The sonopolymer, which is formed from the sonochemical decomposition of the DCB and has been shown to assist exfoliation, was removed from the dispersion by several washing cycles with chloroform as previously described^17^.

### Basic characterization

To measure the concentration of WSe_2_ in dispersion, 25 μl of the dispersion was dried in an aluminium pan, and weighted by Perkin Elmer AD 6 Autobalance. TEM, high-resolution-TEM and SAED were measured by FEI Tecnai Osiris electron microscope. SEM pictures were taken with a Zeiss Merlin microscope. The absorption spectra of the thin films were recorded by using a UV–vis-NIR UV-3600 (Shimadzu) spectrophotometer. Raman spectra were carried out on LabRAM HR Raman spectrometer with 532 nm Laser. W core XPS spectra were acquired with a KRATOS AXIS ULTRA spectrometer (Al Kα source, 600 × 750 μm spot size).

### SCSA and AC film deposition

The thin film deposition was performed with HTS functionalized WSe_2_ dispersion in hexylamine (*ca.* 10 mg ml^–1^) as described in the main text. Typically for the SCSA method, 20 μl of the WSe_2_ dispersion was injected near an interface (area of 16 cm^2^) created from 15 ml of EG and 10 ml of hexane. After the 2D self-assembly, hexane was removed by a pipette and the film was allowed to dry. A low vacuum (∼50 mbar) was used to assist removing EG from the bottom through fritted glass. A single SCSA deposition gave a thickness of *ca.* 10 nm (estimated by SEM), in correspondence with the quantity of WSe_2_ injected (*ca.* 10 μg cm^–2^). For the AC film deposition, 300 μl of the WSe_2_ dispersion was injected on to the EG in the absence of the hexane layer. The amount of the HTS capping agent remaining after the deposition was estimated in the following manner. Two separate one-flake-layer thin films of WSe_2_ were deposited on glass substrates by the SCSA method from a non-HTS-modified WSe_2_ dispersion (not a stable dispersion). After dying at 150 °C, one film was dipped in HTS solution (0.5 vol% in DCB solvent) for 30 min. Meanwhile the control film was dipped in neat DCB for the same time. Then the films were washed by DCB and hexane sequentially. The HTS coverage on the WSe_2_ film was then estimated by XPS quantification based on the following two assumptions. (1) The post-HTS treatment modified only one side of the flakes. (2) The Si signal arising from the substrate is constant since films are considered homogeneous over the area probed by the XPS (600 × 750 μm). We found the atomic ratio (Si/W) for the non-modified sample to be 1.10/1, while this ratio increases to 1.15/1 after HTS treatment. Accordingly, we can roughly estimate that the HTS remaining in the SCSA deposited films is in the order of 10 mol%.

### Conductivity measurements

SCSA and AC films were deposited on bare glass, then two Au top electrodes were deposited using thermal resistance evaporation (Kurt J. Lesker Mini-SPECTROS) to construct a conductive channel (20 or 60 μm in length and 1 or 3 mm in width). The thickness of the SCSA and AC films in the channel were estimated to be *ca.* 10 nm (by SEM) and 150 nm (by profilometery), respectively. The *J*–*V* curves of WSe_2_ films were measured in N_2_ atmosphere in dark using a Keithley Model 2612 A Source Meter.

### PEC measurements

SCSA and AC films were deposited on F:SnO_2_-coated glass substrate (Solaronix). The SCSA electrodes were made of two layers of the active material (thickness: 25–30 nm, see Supplementary Fig. 2). The electrodes were tested in a three-electrode set-up (BioLogic SP-200 potentiostat), with a Pt counter electrode and a Ag/AgCl KCl(sat) reference electrode. The electrolyte was a 1 M H_2_SO_4_ solution in deionized water (pH 0) and the active area of the photoelectrode was 0.26 cm^2^. In a typical LSV acquisition, the voltage applied to the working electrode was swept cathodically (10 mV s^–1^) between 0.6 and 0 V versus RHE under intermittent illumination (simulated 1 sun with a filtered Xe arc lamp, illumination spectrum given in recent work^44^). Stability measurements were conducted by applying a constant voltage to the working electrode and recording the evolution of the photocurrent over time. The platinum electrocatalyst was deposited on the surface of the samples using the same three-electrode set-up with a 2 mM H_2_PtCl_6_ aqueous deposition electrolyte. During the deposition, the sample was under illumination and the voltage was swept cathodically at 10 mV s^–1^ between 0.6 and 0.1 V versus RHE and typically 5–6 mC of current was passed. After the deposition, the sample was rinsed with water and dried with compressed air.

### H_2_ measurements and Faradaic efficiency calculation

For this measurement, the working electrode was placed in a specifically designed, tightly closed PEC cell. The counter and reference electrodes, as well as the electrolyte, were the same as described in the PEC measurement section. A tube carrying Argon was inserted into the cell, with its output positioned to bubble the gas in the electrolyte. A second pipe was used to connect the cell headspace and a Clarus 480 Gas Chromatograph from Perklin Elmer. This allowed for real-time analysis of the composition of the gas produced inside the PEC cell. First, the electrolyte and small overhead space inside the cell were thoroughly purged with Argon (the purging process was monitored by GC analysis). Then the working electrode was set at +0.1 V versus RHE under illumination. After the photocurrent reached a steady-state value, the composition of the output gas flow was analysed by GC measurement. For a photocurrent of 9.39 μA (see Supplementary Fig. 8b), the GC spectrum gave an absolute H_2_ signal of 0.1784. To estimate the Faradaic efficiency of the system, the H_2_ detection system was calibrated using platinum foils as both the working and counter electrodes. For different values of steady state currents (obtained by setting the Pt working electrode at different potentials), the H_2_ signal was measured by the GC. From these couple of values, a calibration curve was obtained (Supplementary Fig. 8a). Assuming Pt produces H_2_ with 100% Faradaic efficiency, and based on the calibration curve, a 9.39 μA photocurrent should correspond to a GC signal of 0.1729. Since the signal we actually detected was 0.1784, the corresponding estimated Faradaic efficiency was 103%, with an estimated relative error of 10%.

## Additional information

**How to cite this article:** Yu, X. *et al.* Self-assembled 2D WSe_2_ Thin Films for Photoelectrochemical Hydrogen production. *Nat. Commun.* 6:7596 doi: 10.1038/ncomms8596 (2015).

## Supplementary Material

Supplementary Information Supplementary Figures 1-9 and Supplementary Table 1

Supplementary Movie 1 The movie shows a real-time side-by-side comparison of the liquid/liquid (SCSA) and air/liquid (AC) WSe2 2D flake self-assembly techniques on a laboratory scale (batch format)
