# Supplementary material for: Self-assembled 2D WSe2 thin films for photoelectrochemical hydrogen production
Source: Nat Commun. 2015 Jul 1;6:7596. doi: 10.1038/ncomms8596 (PMC4506493; doi:10.1038/ncomms8596)
Supplement: Supplementary Information — Supplementary Figures 1-9 and Supplementary Table 1 [file ncomms8596-s1.pdf]

Supplementary Figures

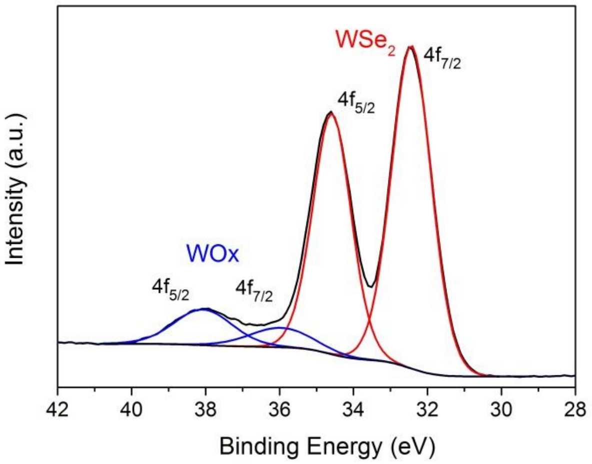

Supplementary Figure 1. The W core level XPS spectrum obtained on a SCSA deposited WSe_2 film.

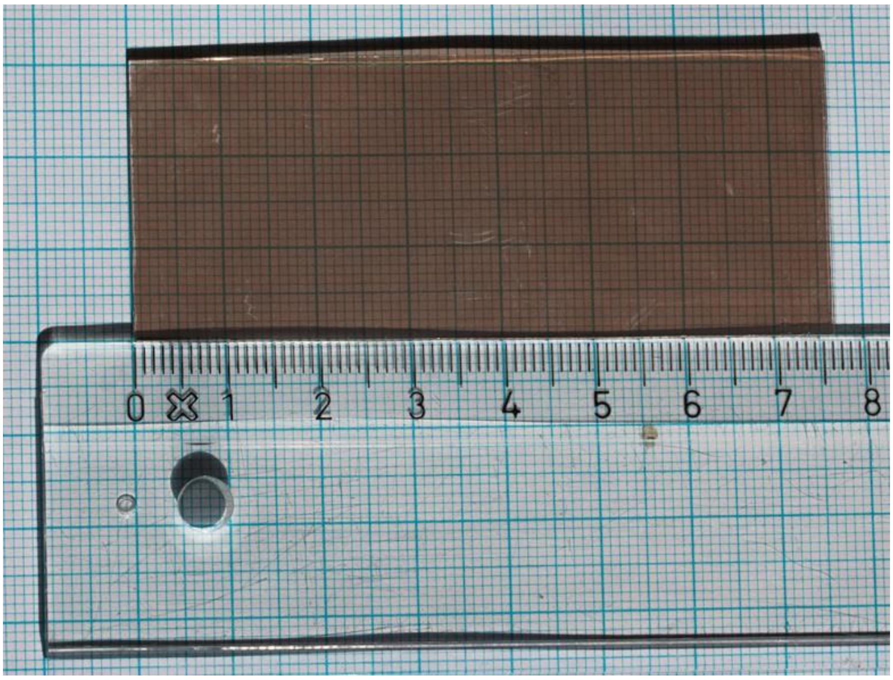

Supplementary Figure 2. A single flake layer WSe_2 film on a glass substrate prepared by the SCSA method. The area of the substrate is 22.5 cm^2. A few scratches are noted on the film due to rough handling.

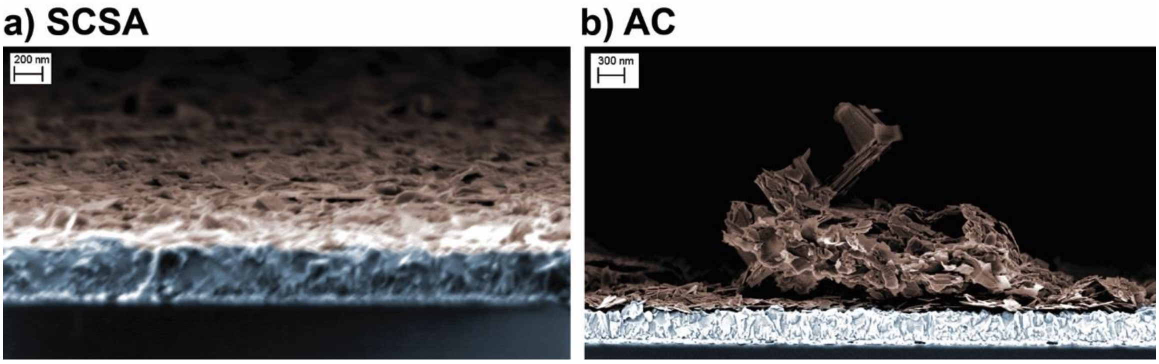

Supplementary Figure 3. Additional cross-sectional SEM images. Panel (a) shows a tilt-shift view (stage at 3°) of the SCSA film to highlight the single-flake nature of the film and the slight roughness induced by the FTO surface. Panel (b) shows a representation of the large aggregates present in the AC film. The SEM images are coloured to highlight the F:SnO_2 substrate (blue) and the WSe_2 (brown).

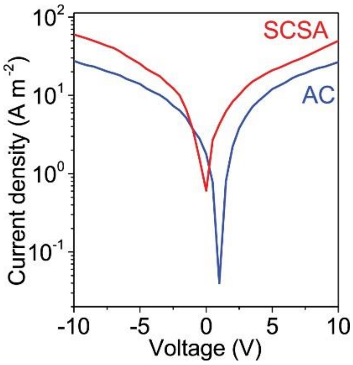

Supplementary Figure 4. The J-V data for the conductivity measurement on the 60 µm channel device as described in the main text. We note the shift of the minimum current by 1 V in conductivity measurement of AC film, which can be attributed to capacitance created during the scan.

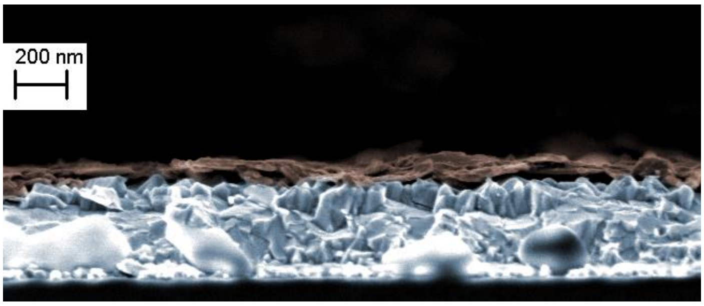

Supplementary Figure 5. Cross-sectional SEM image of a 2 layer SCSA WSe_2 film. A similar morphology is observed as in the 1 layer case (Figure 3c, main text)

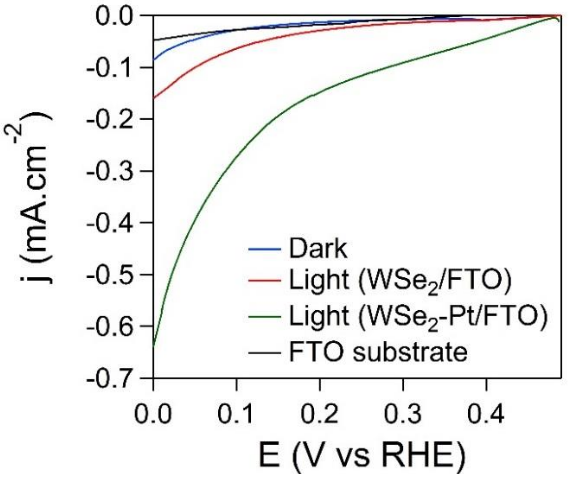

Supplementary Figure 6. Linear scanning voltammetry curves for the WSe_2 under constant dark or illumination conditions. Scan rate 10 mV s^{-1} in 1M H_2SO_4 electrolyte.

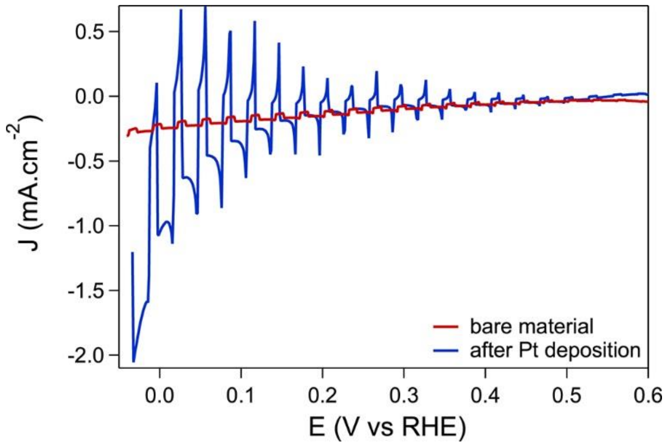

Supplementary Figure 7. Linear scanning voltammetry under intermittent illumination of the best performing 2-layer SCSA WSe_2 film before (red curve) and after (blue curve) Pt deposition.

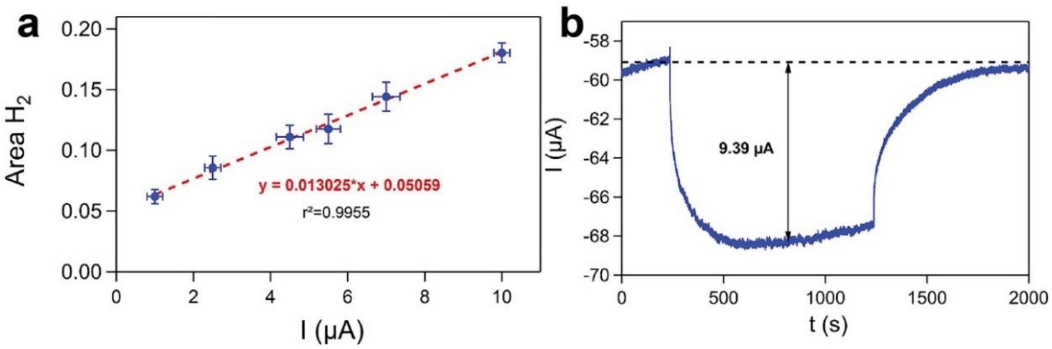

Supplementary Figure 8. H_2 measurements and Faradaic efficiency calculation. (a) Shows the calibration curve constructed using Pt electrodes. (b) Shows the raw photocurrent data from the SCSA WSe_2 photocathode measured.

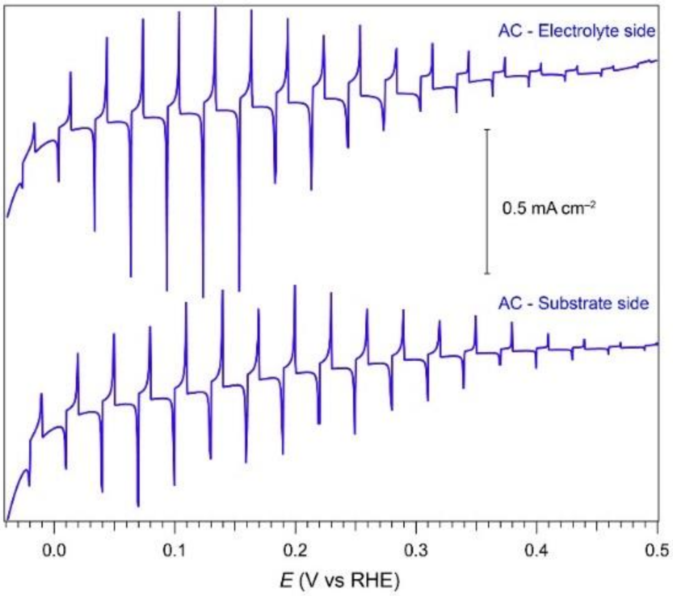

Supplementary Figure 9. Comparison of the linear scanning voltammetry under intermittent 1 sun illumination in 1M H_2SO_4 electrolyte of the Pt-functionalized AC WSe_2 film.

Supplementary Tables

Supplementary Table 1. Summary of conductivity measurements.

**Device geometry**		**Measured conductivity (S m^{-1})**	
**Channel Length (µm)**	**Channel Width (mm)**	**SCSA film**	**AC film**
60	1	6.5×10^{-4}	1.8×10^{-4}
20	3	7.2×10^{-4}	2.4×10^{-4}
